# Effectiveness of prehabilitation during neoadjuvant therapy for patients with esophageal or gastroesophageal junction cancer: a systematic review

**DOI:** 10.1007/s10388-024-01049-9

**Published:** 2024-02-27

**Authors:** Tomohiro Ikeda, Shusuke Toyama, Tsuyoshi Harada, Kazuhiro Noma, Masanori Hamada, Takashi Kitagawa

**Affiliations:** 1 Department of Rehabilitation Medicine, Okayama University Hospital, 2-5-1 Shikatacho, Kita-ku, Okayama, 700-8558 Japan; 2 Department of Rehabilitation, Tagami Hospital, 2-14-15 tagami, Nagasaki, Japan; 3 Department of Rehabilitation Medicine, National Cancer Center Hospital East, 6-5-1 Kashiwa, Chiba, Japan; 4 Department of Rehabilitation Medicine, Keio University Graduate School, 35 Shinanomachi, Shinjuku-ku, Tokyo, Japan; 5 Department of Gastroenterological Surgery, Graduate School of Medicine, Dentistry and Pharmaceutical Sciences, Okayama University, 2-5-1 Shikatacho, Kita-ku, Okayama, Japan; 6 Department of Physical Therapy, School of Health Sciences, Shinshu University, 3‑1‑1 Asahi, Matsumoto, Nagano, Japan

**Keywords:** Prehabilitation, Neoadjuvant therapy, Esophageal cancer, Gastroesophageal junction cancer, Systematic review, Physical weakness

## Abstract

Progression of the physical weakness during neoadjuvant therapy (NAT) in patients with esophageal or gastroesophageal junction cancer is a serious problem; however, prehabilitation during NAT has the potential to overcome the unmet need. Nevertheless, systematic reviews on this topic have not been summarized. Therefore, this systematic review aimed to determine prehabilitation’s effectiveness, acceptability, and safety during NAT for patients with esophageal or gastroesophageal junction cancer. An electronic search was performed in the MEDLINE, Web of Science, CENTRAL, CINAHL, and PEDro databases. A meta-analysis was conducted to assess the effectiveness of prehabilitation during NAT, along with a descriptive analysis of acceptance and safety. This study analyzed data from three randomized controlled trials (RCTs) and nine non-RCTs involving 664 patients. The meta-analysis of two RCTs demonstrated that prehabilitation during NAT may be more effective than usual care in enhancing tolerance to NAT and grip strength; moreover, one RCT and three non-RCTs revealed that prehabilitation may reduce the risk of postoperative complications. The adherence rates for exercise programs in two RCTs and seven non-RCTs were 55–76%. Additionally, two studies reported a 76% adherence rate for multimodal prehabilitation programs, including exercise, dietary, and psychological care. Six studies reported no serious prehabilitation-related adverse events during NAT. Prehabilitation during NAT may be a safe and beneficial intervention strategy for patients with esophageal or gastroesophageal junction cancer. However, the investigation of strategies to enhance adherence is essential. Furthermore, additional high-quality RCTs are needed to examine the effect of prehabilitation during NAT.

**Supplementary Information:**

The online version contains supplementary material available at 10.1007/s10388-024-01049-9.

## Introduction

Esophageal cancer is a common and lethal tumor globally [1]. The standard treatment for locally advanced esophageal or gastroesophageal junction cancers is neoadjuvant therapy (NAT) followed by surgery [2, 3]. Although NAT improves survival compared with surgery alone [2–4], it causes severe adverse events and significantly reduces physical fitness and skeletal muscle mass [5, 6]. In surgeries for esophageal or gastroesophageal junction cancers with significant physical stress, preoperative physical weakness is a severe problem associated with postoperative complications, poor prognosis, and decreased quality of life [7, 8]. Furthermore, the latest studies have shown that themselves, such as reduced physical fitness and skeletal muscle loss during the NAT has a negative impact on postoperative prognosis [6, 9–11]. Therefore, to improve postoperative outcomes, the prevention of physical weakness during NAT is essential, and strategies to achieve this should be prioritized.

Prehabilitation, consisting mainly of aerobic exercise, inspiratory muscle training, and nutritional management, might be feasible and effective for preoperative fitness and clinical outcomes of esophagectomy in the systematic review [12]. Meanwhile, there is a lack of information regarding the effectiveness and feasibility of prehabilitation during NAT in a previous review [12]. Various adverse events and symptoms develop during NAT [3]. For patients undergoing surgery after NAT, a prehabilitation program during NAT tailored to the changes in disease status and physical condition is necessary, distinct from common prehabilitation programs. Several recent studies regarding prehabilitation during NAT for patients with esophageal or gastroesophageal junction cancer have demonstrated the feasibility and effectiveness of prehabilitation on physical fitness, skeletal muscle mass, and tolerance to NAT [13–15]. However, although there are systematic reviews on prehabilitation during NAT in patients with breast and rectal cancer [16, 17], information on clinical practice and evidence gaps in patients with esophageal or gastroesophageal junction cancer have not been summarized.

This systematic review aimed to determine the effectiveness, acceptability, and safety of prehabilitation during NAT for patients with esophageal or gastroesophageal junction cancer.

## Materials and methods

### Search strategy

A literature search was performed on March 10, 2023, to identify relevant studies assessing prehabilitation regimens including exercise in patients with esophageal or gastroesophageal junction cancer during NAT, in the MEDLINE (PubMed), Web of Science, CENTRAL, CINAHL, and Physiotherapy Evidence (PEDro) databases. Unpublished literature was searched using the ClinicalTrials.gov and OpenGrey databases. The search included the following keywords: “esophagus,” “gastric,” “neoadjuvant,” “physical therapy,” “prehabilitation,” and “respiratory training.” The references of the included articles were screened, and a manual search was performed to identify missing articles. The complete electronic search strategy is available in the electronic supplementary material (Online Resource 1). Two reviewers (TI and ST) independently assessed titles and abstracts to include relevant references. Subsequently, the full-text articles selected by title and abstract screening were assessed for eligibility. Data extraction from the included studies was performed independently by two reviewers (TI and ST). In cases of disagreement regarding the inclusion or data extraction, a third author (TK) was consulted. The authors of the included studies were contacted to obtain unpublished data. The Preferred Reporting Items for Systematic Reviews and Meta-Analyses (PRISMA) guidelines [18] were followed (Online Resource 2). The searched references were uploaded to Rayyan [19] (Qatar Computing Research Institute, Ar Rayyan, Qatar), and duplicate references were removed. The protocol for this systematic review was registered with PROSPERO (https://www.crd.york.ac.uk/prospero/display_record.php?ID=CRD42023395998).

### Study selection

We included randomized controlled trials (RCTs) and quasi-RCTs evaluating the feasibility or efficacy of prehabilitation during NAT. Retrospective cohort and single-arm studies were included to investigate the feasibility of this approach. There were no restrictions on the country or language. All published and unpublished papers, including conference abstracts, were eligible. No animal studies were included. No exclusions were made on the basis of the observation period. Systematic reviews, meta-analyses, narrative reviews, cluster RCTs, cross-sectional studies, crossover trials, case series, case reports, and letters were excluded. Studies that met the following criteria were included: those involving patients with esophageal or gastroesophageal junction cancer receiving NAT; those incorporating prehabilitation including aerobic exercise, resistance training, and respiratory training during NAT; and those assessing preoperative physical fitness. Studies that met the following criteria were excluded: participants with unstable comorbidities that precluded exercise therapy or exercise function testing, such as severe cardiac or respiratory dysfunction or orthopedic disease; cognitive disorders (Mini-Mental State Examination score less than 24); and nonradical surgery.

### Assessment outcomes and other variables

The primary outcomes were exercise capacity (cardiopulmonary exercise test and 6-min walk test) and postoperative complications (postoperative pulmonary complications and surgical site infections, etc.). The secondary outcomes included an assessment of physical fitness other than exercise capacity, tolerance to NAT (completion rate of scheduled treatment), the adherence of prehabilitation (attendance/session*100), and adverse events of prehabilitation.

To understand the characteristics of the included studies, we collected information on participants’ characteristics (age, sex, body mass index [BMI], diagnosis, and NAT regimen) and prehabilitation protocols (type, intensity, time, and frequency).

### Assessment for risk of bias and quality of evidence

We assessed the risk of bias in the RCTs using the revised Cochrane risk-of-bias tool for randomized trials (RoB 2) [20]. The RoB2 tool categorizes the risk of bias into “low,” “some concerns,” or “high” groups. The risk of bias in non-randomized comparative studies was assessed using the Newcastle–Ottawa Scale (NOS) [21]. For NOS scores ranging from 0 to 9, we considered scores of 0–3, 4–6, and 7–9 as low, moderate, and high-quality studies, respectively. Single-arm studies were assessed using the National Heart, Lung, and Blood Institute Quality Assessment Tool for before-after (pre- and post-intervention) studies [22]. Based on the 12-item rating, “Good,” “Fair,” and “Poor” scores were determined. All risk-of-bias assessments were conducted in duplicate by two well-trained reviewers (TI and ST). Disagreements between the reviewers were resolved through discussion or by inviting an additional reviewer (TK). The quality of evidence was determined by one researcher (TI) and confirmed and finalized by another researcher (ST). The GRADE (Grading of Recommendations, Assessment, Development, and Evaluation) method was used to check the quality of the evidence [23]. Summary of the findings, prepared according to the Cochrane Handbook is presented in Table 1. Table 1 shows the relative effects per outcome, number of participants, quality of evidence, and additional comments. The standard mean difference (SMD) and risk ratio (RR) were used as relative effects.

**Table 1:** Summary of findings: Effectiveness of prehabilitation during neoadjuvant therapy for patients with esophagogastric cancer

Patient or population: [Preoperative patients with esophagogastric cancer] Setting: [During NAT] Intervention: [Prehabilitation] Comparison: [Usual care]						
Outcomes	Anticipated absolute effects^*^ (95% CI)		Relative effect (95% CI)	№ of participants (studies)	Certainty of the evidence (GRADE)	Comments
	Risk with [Usual care]	Risk with [Prehabilitation]				
Exercise capacity assessed with: Peak VO2/6MWT follow-up: mean 10 weeks	–	SMD 0.93 SD higher ( – 0.30 lower to 2.17 higher)	–	104 (two RCTs)	⨁◯◯◯ Very low	No evidence has shown that prehabilitation during NAT prevents a decline in exercise capacity
Postoperative complication assessed with: CD > IIIa	394 per 1,000	240 per 1,000 (154–370)	RR 0.61 (0.39 to 0.94)	186 (one RCT and three non-RCTs)	⨁◯◯◯ Very low	No evidence has shown that prehabilitation during NAT prevents postoperative complications
Grip strength follow-up: mean 10 weeks	–	SMD 1.22 SD higher (0.34 higher to 1.44 higher)	–	108 (two RCTs)	⨁◯◯◯ Very low	Prehabilitation during NAT may prevent grip strength loss
Skeletal muscle mass assessed with: CT scan follow-up: mean 10 weeks	–	SMD 0.27 SD higher ( – 0.11 lower to 0.65 higher)	–	108 (two RCTs)	⨁◯◯◯ Very low	No evidence has shown that prehabilitation during NAT prevents skeletal muscle loss
Tolerance to NAT assessed with completion rate of scheduled NAT follow-up: mean 10 weeks	482 per 1,000	728 per 1000 (530–1000)	RR 1.51 (1.10 to 2.08)	108 (two RCTs)	⨁⨁◯◯ Low	Prehabilitation during NAT may improve tolerance to treatment
Adherence of prehabilitation follow-up: mean 11 weeks	The adherence rates of prehabilitation programs ranged from 55 to 76%. (In the case of multimodal support, 76%)			418 (two RCTs and seven non-RCTs)	⨁◯◯◯ Very low	Adherence to prehabilitation during NAT may be acceptable in some cases
Adverse events of prehabilitation follow-up: mean 11 weeks	No AE was observed (0%)			423 (two RCTs and four non-RCTs)	⨁◯◯◯ Very low	Prehabilitation during NAT may be safe

GRADE Working Group grades of evidence

High certainty: We are very confident that the true effect is similar to the estimate of the effect

Moderate certainty: We are moderately confident in the effect estimate: the true effect may be similar to the estimate of the effect, with a possibility of substantial differences

Low certainty: Our confidence in the effect estimate is limited: the true effect may substantially differ from the estimate of the effect

Very low certainty: We have very little confidence in the effect estimate: the true effect may substantially differ from the estimate of effect

*CI* confidence interval; *Peak VO*_*2*_ peak oxygen consumption; *6MWT* 6 min walking test; *NAT* neoadjuvant therapy; *RR* risk ratio; *SMD* standardized mean difference; *RCT* randomized controlled trial; *CD* Clavien–Dindo classification; *AE* adverse event

^*^The risk in the intervention group (and its 95% confidence interval) is based on the assumed risk in the comparison group and the relative effect of the intervention (and its 95% CI)

### Statistical analysis

A meta-analysis of the studies comparing the two groups was performed if at least two published RCTs reported the same outcome within the same timeframe. Published non-RCTs and unpublished studies were included in the meta-analysis only when one or fewer published RCTs examined for each outcome. We pooled the mean differences and 95% confidence intervals (CIs) for the continuous variables (reporting mean and standard deviation or standard error of the mean) for each trial. For cases in which the outcome units were different, we attempted to integrate the data by calculating SMD. RRs with 95% CIs were selected as summary statistics for dichotomized outcomes. The meta-analysis was performed using the Review Manager software (RevMan 5.4, Cochrane) [24]. Missing data were referred to the principal investigator, and in cases where they were unavailable, the substitution method was not used. When a meta-analysis was not possible, results were described narratively. In cases with I^2^ > 50%, we assessed substantial heterogeneity, and subgroup analysis was performed by age when we have gathered sufficient data. The Cochran chi-squared test (Q test) was used for the I^2^ statistic, and a p-value of < 0.10 was considered statistically significant. Considering that the intervention effects varied owing to setting differences across studies, we applied a random-effects model. If the mean differences and 95% CIs were not reported, the study was excluded from the meta-analysis. To confirm the robustness of the results, a meta-analysis was performed using non-RCTs for sensitivity analysis. We assessed the possibility of reporting bias using funnel plots.

## Results

### Study identification

**Fig. 1:**
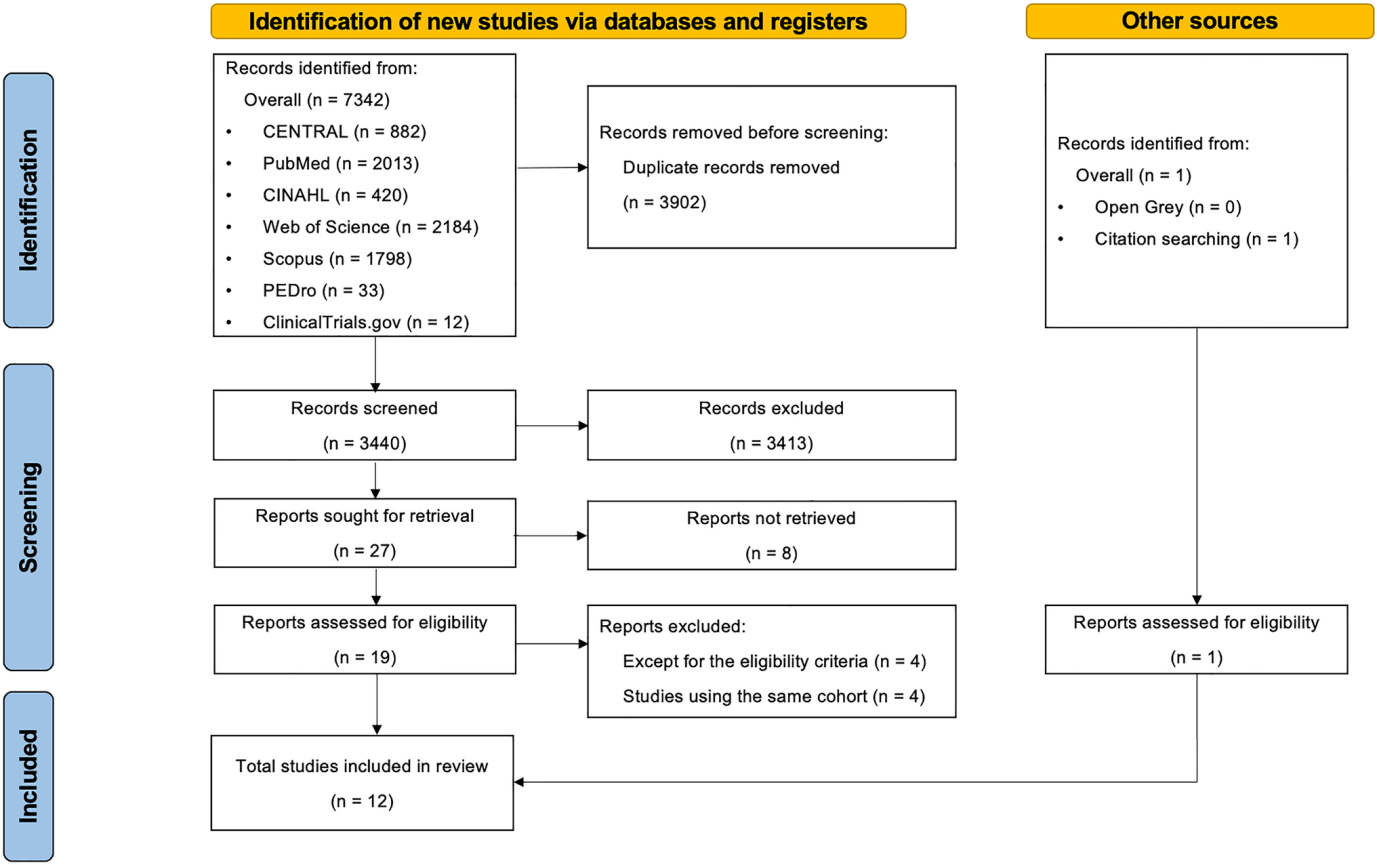
PRISMA flow diagram of study selection. This flow diagram demonstrates the study screening and selection process. *PRISMA* Preferred Reporting Items for Systematic Reviews and Meta-Analyses

A systematic search of the databases yielded 7342 results. Duplicates were removed, leaving 3,902 records. In the primary screening, 3440 articles were reviewed by title and abstract, and in the secondary screening, 27 articles were evaluated for eligibility using the full text. Ultimately, 12 articles [13–15, 25–33] were eligible for inclusion. A systematic search of these databases is shown in Fig. 1.

### Study characteristics

The study characteristics are summarized in Table 2. Three studies were included in each category: RCTs [13, 14, 25], quasi-RCTs [15, 26, 27], retrospective cohort studies [28–30], and prospective single-arm intervention studies [31–33]. The literature consisted of nine original articles [13–15, 26, 28–30, 32, 33] and three unpublished studies [25, 27, 31]. The total number of participants in the 12 included studies was 664. The mean age of the participants ranged from 59.2 to 67.0 years, 74.7 to 100% were men, and the mean BMI ranged from 21.1 to 28.9 kg/m2. Three studies [13, 26, 33] used neoadjuvant chemoradiotherapy, five [15, 27, 29, 30, 32] used neoadjuvant chemotherapy, and three [14, 28, 31] used neoadjuvant chemoradiotherapy or chemotherapy.

**Table 2:** The characteristics of the study and participants

Author	Country	Study design	Article type	Group (n)	Study population
Xu et al. (2015) [13]	China	RCT	Original Article	PG (28) CG (28)	Patients with esophageal cancer undergoing nCRT^b^; 59.6 (9.3) years old; 92.9% men; body weight was 58.5 (9.4) kg; 82.1% were in clinical stage III
Allen et al. (2022) [14]	UK	RCT	Original Article	PG (26) CG (28)	Patients with esophagogastric cancer undergoing nCT or nCRT; 64 (8) years old; 85.2% men; BMI 27.9 (4.9) kg/m^2^; AJCC^a^ pathologic stages T3–4 were 75.9% and N2–3 were 35.1%
Loughney et al. (2021) [25]	Ireland	RCT	Abstract	PG (36) CG (35)	Patients undergoing neoadjuvant cancer treatment and surgical resection for esophagogastric malignancies; age: NA; sex: NA; BMI: NA; clinical or pathologic stage information: NA
Zylstra et al. (2022) [15]	UK	quasi-RCT	Original Article	PG (22) CG (20)	Patients with lower esophageal or gastroesophageal junction cancer undergoing nCT; 62.0 (9.5) years old; 85.7% men; BMI 28.9 (6.1) kg/m^2^; clinical stages T3–4 were 90% and N2–3 were 40%
Christensen et al. (2018) [26]	Denmark	quasi-RCT	Original Article	PG (27) CG (35)	Patients with cancer of the gastro‐esophageal junction undergoing nCRT; 64.8 (7.7) years old; 90% men; BMI 28.1 (5.5) kg/m^2^; 30% were in clinical stage III
Knight et al. (2022) [27]	UK	quasi-RCT	Abstract	PG (22) CG (20)	Patients who received a structured prehabilitation exercise program before and after nCT^c^ or surgery for esophageal cancer; age: NA; sex: NA; BMI: NA; clinical or pathologic stage information: NA
Halliday et al. (2023) [28]	UK	cohort	Original Article	PG (51) CG (28)	Patients who underwent esophagectomy after nCT or nCRT for esophageal or gastroesophageal junction cancer; 65.2 (9.8) years old; 74.7% men; BMI 27.2 (5.6) kg/m^2^; 84.8% were clinical stages III–IV
Ikeda et al. (2022) [29]	Japan	cohort	Original Article	PG 1 (39) PG 2 (71)	Patients with esophageal cancer undergoing nCT; 65.4 (8.9) years old; 83.6% men; BMI 21.1 (3.0) kg/m^2^; 77.2% were in clinical stage III–IV
Halliday et al. (2021) [30]	UK	cohort^d^	Original Article	PG (60)	Patients with esophageal or gastroesophageal junctional adenocarcinoma undergoing nCT; 66 (9.7) years old; sex: NA; BMI: NA; clinical stages T3–4 were 76.1% and N2–3 were 16.4%
Kenneth (2021) [31]	France	single-arm	Thesis	PG 1 (5) PG 2 (2) PG 3 (6)	Patients who underwent esophagectomy after nCT or nCRT for esophageal cancer; 60.5 (9.6) years old; 76.9% men; BMI 29.8 (4.9) kg/m^2^; 76.9% were AJCC pathological stages III–IV
Chmelo et al. (2022) [32]	UK	single-arm	Original Article	PG (39)	Patients with locally advanced esophageal and gastric adenocarcinoma receiving nCT; 67.0 (7.0) years old; 84.6% men; BMI 28.9 (5.0) kg/m^2^; clinical or pathologic stage information: NA
Yang et al. (2021) [33]	Korea	single-arm	Original Article	PG (36)	Patients with esophageal cancer receiving nCRT; 59.2 (6.5) years old; 100% men; BMI 22.9 (2.3) kg/m^2^; 70.0% were clinical stages T3–4 and 40.0% were N2–3

*NA* not available. *PG* Prehabilitation group; CG Control group

^a^AJCC, American Joint Committee of cancer

^b^nCRT, neoadjuvant chemoradiotherapy

^c^nCT, neoadjuvant chemotherapy

^d^Characteristics of the cohort including six patients with non-NAT

### Characteristics of the prehabilitation intervention

The characteristics of prehabilitation during NAT are presented in Table 3. Prehabilitation programs mainly consisted of aerobic exercise and resistance training, and the duration ranged 4–16 weeks, with one to seven sessions per week. Aerobic exercise duration ranged 15–60 min, performed at low to moderate intensity [13, 14, 29, 32, 33] moderate/vigorous-intensity activity [28, 30], or high-intensity interval training [15, 26, 31]. Aerobic exercise included programs with expert instruction [13–15, 26, 29, 31], self-training [28–33], and remote communication using phone calls or applications [28, 30–33]. Resistance training was performed at moderate to high intensity with expert instruction [15, 26, 29] or self-training [14, 28–32]. Additionally, some studies incorporated nutritional interventions [13, 14, 28–31, 33] and medical counseling [14, 29–31] within their prehabilitation programs.

**Table 3:** The characteristics of the intervention and measured outcomes

Study	Study period	Prehabilitation group	Control group	Measured outcomes
Xu et al. (2015) [13]	NAT	Aerobic exercise: supervised walking sessions during NAT (4–5 weeks; three sessions/week; 25 min/day; moderate intensity) Resistance training: no sessions Others: nutritional guidance weekly during the intervention period	All patients, including the control group, received regular nutrition and self-care advice from staff nurses before initiating radiation therapy	Exercise capacity (6MWT), muscle strength (HGS), treatment tolerance, adverse event of NAT, adherence
Allen et al. (2022) [14]	NAT, post-NAT	Aerobic exercise: supervised sessions during NAT (15 weeks; two sessions/week; 60 min/day; moderate intensity) Resistance training: self-exercise sessions during NAT (15 weeks; three sessions/week; 60 min/day; moderate intensity) Others: nutrition guidance and psychological counseling during NAT (total six sessions)	All patients, including the control group, were instructed by physicians and nurses to improve physical activity, receive individualized dietary guidance, and wear activity trackers	Exercise capacity (peakVO_2_), muscle strength (HGS), skeletal muscle mass, HRQOL (EORTC QLQ-C30), postoperative complication, length of hospital stays, cancer-related mortality, adherence to intervention, drop out from intervention
Loughney et al. (2021) [25]	NAT, post-NAT	NA	NA	Exercise capacity (6MWT), physical performance
Zylstra et al. (2022) [15]	NAT	Aerobic exercise: supervised sessions during NAT (4 weeks; five sessions/week; 30 min/day; HIIT) Resistance training: supervised session during NAT (core strength and band strength exercises; moderate intensity) Others: Stability and flexibility exercise	No restrictions on physical activity were imposed on the control group	Fat-free mass, postoperative complication, length of hospital stays, failed scheduled treatment, tumor regression, drop out from intervention
Christensen et al. (2018) [26]	NAT, post-NAT	Aerobic exercise: supervised sessions during NAT (12 weeks; two sessions/week; 15–20 min/day; HIIT) Resistance training: supervised sessions during NAT (12 weeks; two sessions/week; three sets of 8–12 repetitions; 50–80% 1RM) Others: No special note	In the control group, patients were allowed exercise sessions provided by the hospital or municipality and followed standard treatment	Exercise capacity (peakVO_2_), muscle strength, lean body mass, postoperative complication, length of hospital stays, failed scheduled treatment, HRQOL, adherence to intervention, drop out from intervention, adverse event of intervention
Knight et al. (2022) [27]	NAT	NA	NA	Exercise capacity (peakVO_2_), lean body mass, HRQOL
Halliday et al. (2023) [28]	NAT, post-NAT	Aerobic exercise: self-exercise session with weekly telephone guidance during NAT (16 weeks; 150 min/week; moderate to hard) Resistance training: self-exercise session with weekly telephone guidance during NAT (16 weeks) Others: nutrition interventions based on ESPEN guidelines and weekly or fortnightly telephone calls were used to monitor nutritional status	The control group did not receive prehabilitation and received equivalent perioperative care	Muscle strength (HGS), postoperative complication, skeletal muscle index, adherence to intervention, drop out from intervention
Ikeda et al. (2022) [29]	NAT, post-NAT	Aerobic exercise: supervised sessions during NAT (4 weeks; five sessions/week; 20–40 min/day; low to moderate intensity) and self-exercise sessions after NAT (4 weeks; five sessions/week; 20–40 min/day; low to moderate intensity) Resistance training: supervised sessions during NAT (4 weeks; 2–3 sessions/week; 5–20 min/day; low to hard intensity) and self-exercise sessions after NAT (4 weeks; 2–3 sessions/week; 5–20 min/day; low to hard intensity) Others: multimodal interventions, including psychological care, nutritional guidance, and oral care (4 weeks)	No control group	Skeletal muscle mass, tumor regression, postoperative complication, adherence to the intervention, adverse event of intervention and NAT
Halliday et al. (2021) [30]	NAT, post-NAT	Aerobic exercise: self-exercise sessions with weekly telephone guidance during NAT (16 weeks; 150 min/week; moderate to hard) Resistance training: self-exercise sessions with weekly telephone guidance during NAT (16 weeks) Others: patients received telephone consultation from a clinical nurse specialist as needed	No control group	Exercise capacity (peakVO_2_), postoperative complication, length of hospital stays, adherence to intervention
Kenneth (2021) [31]	NAT, post-NAT	Aerobic exercise: hospital-based or virtual home-based supervised sessions (16 weeks; two sessions/week; 28 min/day; HIIT) or self-exercise sessions (16 weeks; three sessions/week; 30 min/day; moderate intensity) Resistance training: self-exercise sessions (16 weeks; eight exercises; two sessions/week; 2–3 sets of 8–12 repetitions; hard intensity) Others: respiratory training (30% of PImax; six sets of 10 repetitions); nutritional support with whey-based supplements, psychological support at least one consultation	No control group	Exercise capacity (peakVO_2_ and 6MWT), muscle strength (HGS and QS), HRQOL, adverse event of intervention and NAT, adherence to the intervention, drop out from intervention
Chmelo et al. (2022) [32]	NAT, post-NAT	Aerobic exercise: self-exercise sessions with weekly telephone guidance during NAT (12–15 weeks; seven sessions/week; 30 min/day; moderate intensity) Resistance training: self-exercise sessions with weekly telephone guidance during NAT (12–15 weeks; seven sessions/week; 10 min/day) Others: No special note	No control group	Exercise capacity (peakVO_2_), muscle strength (HGS), lean body mass, respiratory function (FEV1 and FVC), adherence to intervention, drop out from intervention, HRQOL
Yang et al. (2021) [33]	NAT	Aerobic exercise: self-exercise sessions using the application during NAT (8 weeks) Resistance training: no sessions Others: providing feedback messages and encouragement through application by nutrition and exercise therapy specialists	No control group	Skeletal muscle index, adherence to intervention, drop out from intervention

*NAT* neoadjuvant therapy; *6MWT* 6 min walking test; *HGS* hand grip strength; *peakVO*_*2*_ peak oxygen consumption; *HRQOL* health-relate quality of life; *EORTC QLQ-C30* The European Organization for Research and Treatment of Cancer QLQ-C30; *NA* not available; *HITT* high-intensity interval training; *RM* repetition maximum; *QS* quadriceps strength; *FEV1* Forced expiratory volume 1 s; *FVC* forced vital capacity; *PImax* maximal inspiratory pressure; *PEmax*, maximal expiratory pressure

### Assessment of risk of bias

**Fig. 2:**
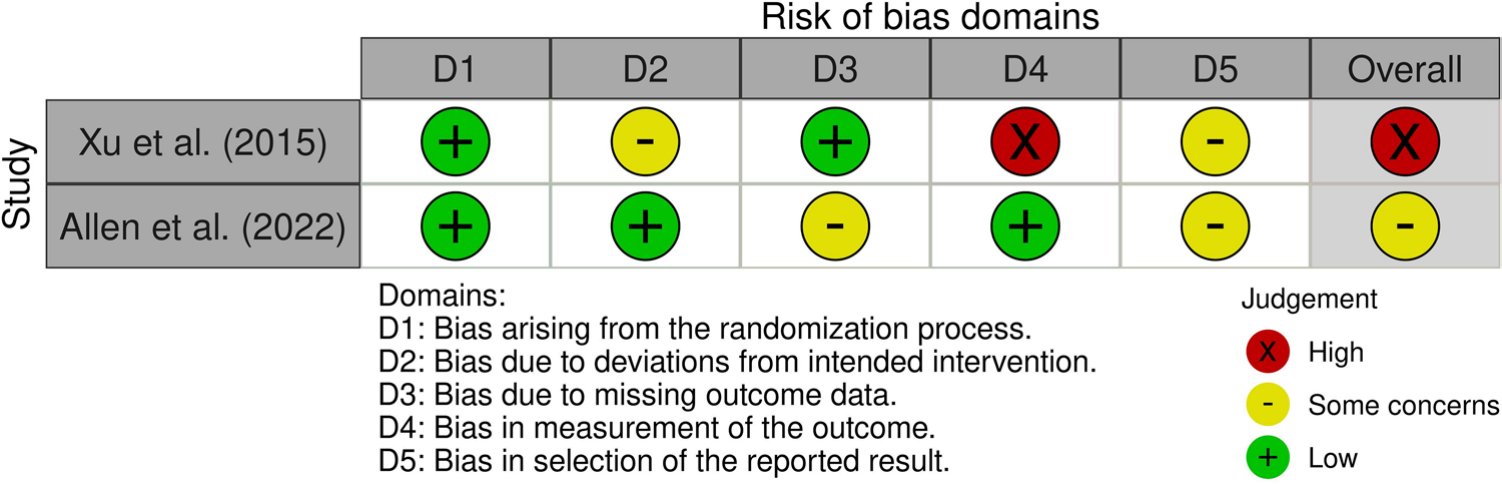
Results of bias risk assessment of RCTs using RoB 2. The risk of bias in randomized controlled trials (RCTs) is assessed using risk of bias 2 (RoB2) at three levels: low, some concerns, and high

The risk of bias was assessed only for original articles. We presented the risk of bias assessment for exercise capacity as the primary outcome of the published two RCTs (Fig. 2). The bias risks for the two RCTs [13, 14] were categorized as “some concerns” and “high,” respectively. The bias in the outcome measurement was judged to be “high” for one RCT [13]. Three comparative studies, excluding RCTs, were judged to be of “high” [15, 28] or “moderate” quality [26] using the NOS (Online Resource 3). In comparison, one non-RCT [26] had a risk of bias. Two non-RCTs [15, 28] had a risk of bias in the outcome assessment. Four single-arm studies were judged to be of good [29] or fair [30, 32, 33] quality (Online Resource 4). Three studies [30, 32, 33] did not report the blinding of outcome assessments, suggesting the possibility of lower quality.

### Effect of prehabilitation

**Fig. 3:**
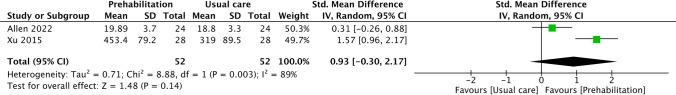
Effect of prehabilitation during NAT on exercise capacity. Forest plot shows effect on exercise capacity after NAT. Forest plot using only randomized controlled trials. *Std.* standard; *95% CI* 95% confidence interval; *NAT* neoadjuvant therapy. Cases with I^2^ > 50% are considered substantially heterogeneous

**Fig. 4:**
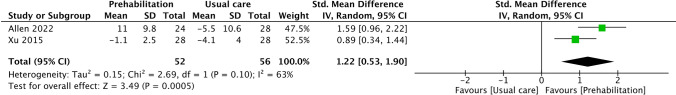
Effect of prehabilitation during NAT on the change of grip strength. Forest plot using only randomized controlled trials. *Std.* standard; *95% CI* 95% confidence interval; *NAT* neoadjuvant therapy. Cases with I^2^ > 50% are considered substantially heterogeneous

**Fig. 5:**
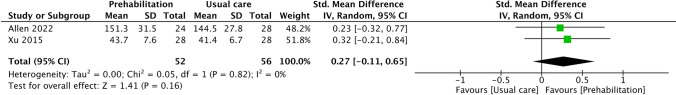
Effect of prehabilitation during NAT on skeletal muscle mass. Forest plot shows effect on skeletal muscle mass after NAT. Forest plot using only randomized controlled trials. *Std.* standard; *95% CI* 95% confidence interval; *NAT* neoadjuvant therapy. Cases with I^2^ > 50% are considered substantially heterogeneous

**Fig. 6:**
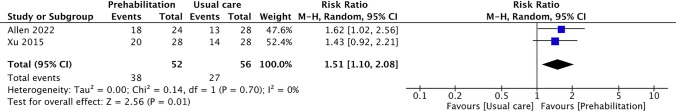
Effect of prehabilitation on tolerance to NAT. Forest plot shows effect on NAT completion. Forest plot using only randomized controlled trials. *95% CI* 95% confidence interval; *M-H* Mantel–Haenszel; *NAT* neoadjuvant therapy. Cases with I^2^ > 50% are considered substantially heterogeneous

**Fig. 7:**
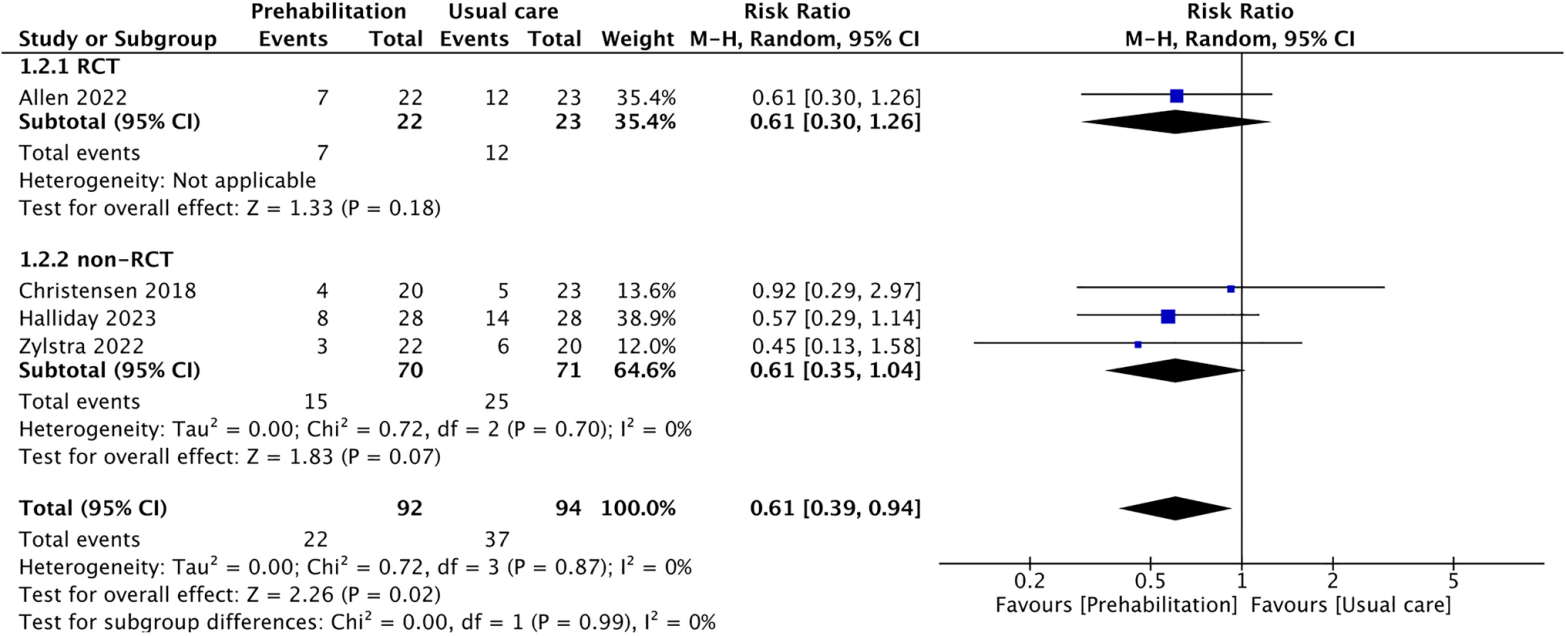
Effect of prehabilitation during NAT on postoperative complications. Forest plot using randomized controlled trial (RCT) and non-RCTs. *95% CI* 95% confidence interval; *M-H* Mantel–Haenszel; *NAT* neoadjuvant therapy. Cases with I^2^ > 50% are considered substantially heterogeneous

Forest plots using two RCTs [13, 14] are shown for the effects of prehabilitation during NAT on exercise capacity (Fig. 3), grip strength (Fig. 4), skeletal muscle mass (Fig. 5), and tolerance to NAT (Fig. 6). Only one RCT [14] assessed the postoperative complications; hence, meta-analysis was performed using non-RCTs [15, 26, 28] (Fig. 7). In all four studies [14, 15, 26, 28], complications were assessed with Clavien–Dindo classification of 3 or higher. A summary of the effects of prehabilitation during NAT is shown in Table 4. Prehabilitation affected completion rate of NAT (RR [95% CI], 1.51 [1.10, 2.08]) and the change in grip strength during NAT (SMD [95% CI], 1.22 [0.53, 1.90]). However, prehabilitation have no significant effect on exercise capacity (SMD [95% CI], 0.93 [-0.30, 2.17]) and skeletal muscle mass (SMD [95% CI], 0.27 [ – 0.11, 0.65]). Prehabilitation during NAT reduced the risk of postoperative complications (RR [95% CI], 0.61 [0.39, 0.94]). In the sensitivity analysis, including RCTs, non-RCTs, and unpublished literature [13–15, 25], prehabilitation during NAT had a significant effect on the exercise capacity (SMD [95% CI], 1.63 [0.11, 3.14]; Online Resource 5) and skeletal muscle mass (SMD [95% CI], 0.36 [0.02, 0.70]; Online Resource 6). Due to the small number of RCTs, a subgroup analysis could not be performed.

**Table 4:** Main findings on the effectiveness and feasibility of prehabilitation

Author	Group	Study period	Physical fitness	Clinical outcomes	Feasibility of prehabilitation
Xu et al. (2015) [13]	PG/CG	NAT	6MWT (vs. CG): ↑ HGS (vs. CG): ↑ SMM (vs. CG): NS	Tolerance to NAT (vs. CG): ↑	Adherence: 68.0% (supervised exercise) and 100% (dietary) Drop out: 0% Adverse event: 0%
Allen et al. (2022) [14]	PG/CG	NAT, post-NAT	Peak VO2 (vs. CG): ↑ HGS (vs. CG): ↑ SMM (vs. CG): NS	QOL (vs. CG): ↑ Postoperative course (vs. CG): NS Tolerance to NAT (vs. CG): ↑	Adherence: 76.0% (supervised exercise), 65.0% (home-based exercise), and 82.0% (psychological care) Dropout: 7.7% (n = 1: travel issues, n = 1: chemotherapy-related arterial thrombus) Adverse events: 0%
Loughney et al. (2021) [25]	PG/CG	NAT, post-NAT	6MWT (vs. CG): ↑	NA	NA
Zylstra et al. (2022) [15]	PG/CG	NAT	SMM (vs. CG): ↑	Tumor regression (vs. CG): ↑ Tolerance to NAT (vs. CG): NS Postoperative course (vs. CG): NS	NA
Christensen et al. (2018) [26]	PG/CG	NAT, post-NAT	Peak VO2 (vs. pre-NAT): ↑	Tolerance to NAT (vs. CG): NS Postoperative course (vs. CG): NS	Adherence: 68.7% (supervised exercise) Dropout: 14.3% (n = 2: health-related problems, n = 1: non-health problem) Adverse events: 0%
Knight et al. (2022) [27]	PG/CG	NAT	Peak VO2 (vs. CG): ↑	NA	NA
Halliday et al. (2023) [28]	PG/CG	NAT, post-NAT	SMM (vs. CG): ↑	Postoperative course (vs. CG): NS	Adherence: 55.0% (home-based exercise) Dropout: 0% Adverse events: NA
Ikeda et al. (2022) [29]	PG	NAT, post-NAT	NA	NA	Adherence: 76.1% (supervised exercise) Dropout: 0% Adverse events: 0%
Halliday et al. (2021) [30]	PG	NAT, post-NAT	NA	NA	Adherence: 56.0% (home-based exercise) Dropout: 0% Adverse events: NA
Kenneth (2021) [31]	PG	NAT, post-NAT	NA	NA	Adherence: 59.6% (supervised exercise), 61.9% (home-based exercise), 80.8% (dietary) Dropout: 0% Adverse events: 0%
Chmelo et al. (2022) [32]	PG	NAT, post-NAT	NA	NA	Adherence: 64.8% (home-based exercise), 98.7% (wearing a pedometer), 100.0% (weekly telephone psychological care) Dropout: 7.7% (n = 2: difficult to implement prehabilitation in parallel with treatment, n = 1: found prehabilitation takes time) Adverse events: 0%
Yang et al. (2021) [33]	PG/CG	NAT	SMM (vs. CG): ↑	NA	Adherence: 69.4% (home-based exercise) Dropout: 2.6% (n = 1: non-use of application) Adverse events: NA

*NA* Not available; *PG* Prehabilitation group; *CG* Control group; ↑ significantly better (p < 0.05); *NS* no significant difference; *NA* not available; *6MWT* 6 min walking test; *HGS* hand grip strength; *peakVO2* peak oxygen consumption; *HRQOL* health-related quality of life; *NAT* neoadjuvant therapy; *Adherence* attendance/sessions × 100

### Feasibility of the prehabilitation

Feasibility of the prehabilitation during NAT is presented in Table 4. Five studies [13, 14, 26, 29, 31] reported adherence (attendance/sessions*100) to supervised exercise during NAT, ranging 59.6–76.1%. Adherence to low- to moderate- intensity supervised exercise included in multimodal prehabilitation during NAT was 76.0% or 76.1% [14, 29]. Six studies [14, 28, 30–33] reported 55.0–69.4% adherence to home-based exercises. Adherence to home-based exercises using the application was relatively high (69.4%) [33]. Two studies [13, 31] reported 80.8–100% adherence to dietary interventions. Two studies [14, 32] reported 82.0–100% adherence to psychological care. Nine studies [13, 14, 26, 28–33] reported 0–14.3% dropout rates of participants with no disease association in the experimental group. Dropout rates from low- to moderate-intensity supervised exercise included in multimodal prehabilitation during NAT were relatively low (0–7.7%). The characteristics of dropouts from prehabilitation included health-related and non-health concerns (travel, lack of time, and lack of energy) [14, 26, 32, 33]. Six studies [13, 14, 26, 29, 31, 32] reported no serious adverse events (0%).

## Discussion

### Summary of main results

This systematic review, encompassing data from 12 articles involving 664 participants, aimed to assess the effectiveness, acceptability, and safety of prehabilitation during NAT for patients with esophageal or gastroesophageal junction cancer. The meta-analysis, including two RCTs, demonstrated that prehabilitation was more effective than usual care regarding tolerance to NAT and grip strength. Moreover, an extensive meta-analysis including RCTs, non-RCTs, and unpublished literature showed that prehabilitation during NAT might contribute to a decrease in the risk of postoperative complications and the maintenance of exercise capacity and skeletal muscle mass. Multimodal prehabilitation approaches, including supervised exercise and dietary and psychological care, were well accepted. Serious adverse events related to prehabilitation were not reported.

### Implications of the present review

To our knowledge, this study is the first systematic review of the effectiveness, acceptability, and safety of prehabilitation for patients with esophageal or gastroesophageal junction cancer during NAT. Although prehabilitation is considered a standard approach before major surgeries and several reviews have reported its efficacy, acceptability, and safety information on NAT was lacking [12]. Furthermore, a previous review [34] of prehabilitation during NAT for all types of cancer has not focused on important RCTs in patients with esophageal cancer [13, 14, 25]. During NAT, patients with esophageal or gastroesophageal junction cancer experience medical problems such as malnutrition [35] and mental illness [36] due to treatment toxicity. Therefore, we conducted a systematic review of prehabilitation during NAT to summarize the current evidence to develop prehabilitation program specifically for patients during NAT and presented three main findings. First, a meta-analysis of two RCTs [13, 14] showed an effect of prehabilitation on tolerance to NAT and a change in grip strength during NAT. Previous reviews [37, 38] have shown that frailty, namely reduced physiological reserve, leads to vulnerability to the stress of cancer treatment. Therefore, prehabilitation during NAT might contribute to preventing frailty for NAT and surgery against esophageal or gastroesophageal junction as part of multimodal cancer treatment. Second, one RCT and three non-RCTs [14, 15, 26, 28] showed that prehabilitation may reduce the risk of postoperative complications. This finding is essential because postoperative complications after esophagectomy are recognized as one of the most critical concerns. Third, prehabilitation during NAT may be considered safe, showing no significant adverse events related to prehabilitation, irrespective of its frequency, intensity, time, or type. Nevertheless, investigating strategies to enhance adherence to prehabilitation programs is essential. The sample size and number of RCTs are insufficient to make specific recommendations for prehabilitation of patients with esophageal or gastroesophageal junction cancer during NAT; however, these findings will guide the development of future prehabilitation programs.

### Effect of prehabilitation during NAT

#### Physical fitness

Our meta-analysis using only RCTs demonstrated a preventive effect of prehabilitation on the grip strength loss during NAT (SMD [95% CI], 1.22 [0.53, 1.90]). Furthermore, meta-analyses that included RCTs, non-RCTs, and unpublished literature demonstrated preventive effects on the exercise capacity (SMD [95% CI], 1.63 [0.11, 3.14]) and skeletal muscle mass loss (SMD [95% CI], 0.36 [0.02, 0.70]). A meta-analysis using only RCTs showed an SMD [95% CI] of 0.93 [ – 0.30, 2.17] for exercise capacity and 0.27 [ – 0.11, 0.65] for skeletal muscle mass, indicating that a prevention effect could be determined in the future with a sufficient sample size. The results are consistent with the evidence in patients with breast cancer [16] and are considered valid. Since physical fitness indices are key surrogate markers of postoperative clinical outcomes, our findings regarding the effects of prehabilitation during NAT are valuable.

#### Tolerance to NAT

Interestingly, a meta-analysis of the present review showed that prehabilitation during NAT might be effective in completion rate of scheduled NAT (RR [95% CI], 1.51 [1.10, 2.08]). Xu et al. [13] showed a trend of higher completion rate of NAT in the prehabilitation group than in the usual care group (71% vs. 50%), and Allen et al. [14] found higher completion rates of NAT at full dose (75% vs. 46%). Previous studies have shown that sarcopenia, namely skeletal muscle loss and muscle weakness/physical function decline, in patients with cancer may impact treatment discontinuation and dose reduction [39–41]. Furthermore, our meta-analysis suggests that prehabilitation during NAT may have a positive impact on grip strength, and sensitivity analysis showed that prehabilitation affected exercise capacity and skeletal muscle mass. Therefore, prehabilitation may have enhanced the tolerability of NAT by preventing sarcopenia. The effect of prehabilitation on tolerance to NAT may contribute to improving dysphagia, minimizing surgical invasion through tumor regression, reducing postoperative recurrence by completing a standard course of NAT, and maximizing the therapeutic benefit [2, 3]. Therefore, evaluation of the impact of prehabilitation during NAT on tolerance to NAT and long-term outcomes should be a priority for future clinical trials.

#### Postoperative complications

The meta-analysis including one RCT and three non-RCTs suggested that prehabilitation during NAT may reduce the risk of postoperative complications. Prehabilitation during NAT may have contributed to the reduction of postoperative complications through the improvement in exercise capacity, grip, and skeletal muscle mass, which are risk factors [6, 9–11]. The results of our meta-analysis on the potential effectiveness of prehabilitation during NAT on physical fitness indices support this hypothesis. This finding is essential because postoperative complications after esophagectomy are one of the most acknowledged critical concerns. However, the evidence is insufficient, and further RCTs should be conducted to clarify the impact of prehabilitation during NAT on the postoperative complications.

### Feasibility of prehabilitation during NAT

A previous study [42] demonstrated that prehabilitation was effective in enhancing exercise capacity for patients with rectal cancer during NAT. This effective program was characterized by an excellent adherence rate of 96% [42]. Consequently, this review focused on identifying a prehabilitation program that would be acceptable for patients with esophageal cancer during NAT as a secondary outcome. Considering most reports conventionally consider ≥ 75% adherence as acceptable [43], in our review, attendance for dietary and psychological care was commendable, exercise attendance was 56–76%, with inadequate and acceptable programs. Notably, participation rates were acceptable (76%) [14, 29] for low- to moderate-intensity supervised exercise included in multimodal prehabilitation, with low dropout rates due to health-related or other reasons (0–7.7%), suggesting better feasibility. As patients with esophageal or gastroesophageal junction cancer experience multiple medical problems during NAT, multimodal interventions [14, 29] may be beneficial to promote adherence to prehabilitation. Previous studies [44–46] theoretically indicated the importance of combining exercise therapy with nutritional and psychological care, supporting our hypothesis. Additionally, multilevel intensity exercise [47] and intensive monitoring may be needed for patients with disincentive factors such as baseline fitness weakness or poor motivation [48]. As for prehabilitation programs involving unsupervised or high-intensity exercise, adherence tends to be insufficient; thus, innovations for enhancing feasibility are warranted.

This review examined the safety of various evidence-based prehabilitation programs. Prehabilitation during NAT may be safe, showing no significant adverse events related to prehabilitation, irrespective of its frequency, intensity, time, or type. Previous reviews found exercise therapy during chemotherapy, including NAT, is generally safe for patients with cancers other than esophageal or gastroesophageal junction cancer [16, 17, 49]. Our review supports this, suggesting that prehabilitation during NAT for patients with esophageal or gastroesophageal junction cancer may also be safe.

### Quality of evidence

The quality of the studies included in this review ranged from low to high; therefore, the results must be interpreted with caution. Specifically, we found that both RCTs and non-RCTs have a high risk of bias in the measurement of outcomes. This issue needs to be addressed in future clinical trials. Three RCTs were conducted, all of which had small sample sizes, raising concerns regarding imprecision. To determine the effectiveness and feasibility of prehabilitation during the NAT period, a meta-analysis should be conducted using clinical trials with larger sample sizes.

### Limitations

This systematic review had several limitations. First, there was heterogeneity in the NAT regimens. Second, there is insufficient RCTs regarding its effect on clinical outcomes. Future studies may reveal additional benefits for postoperative clinical outcomes, considering the potential effectiveness of prehabilitation during NAT. Third, we could not examine what composition would be best for multimodal prehabilitation during NAT. In recent years, multimodal supportive care for patients with advanced cancer are becoming the standard, and additional studies are needed to determine optimal combinations of multiple components. Fourth, the small number of included studies made it difficult to accurately assess reporting bias using funnel plot. Fifth, the sample sizes of the two RCTs are small, the intervention methods vary, and the results differ from the results of the sensitivity analysis, thus the robustness of the results is weak. Finally, due to the small sample size, we have not conducted a subgroup analysis to examine the impact of age on the effectiveness of prehabilitation. Considering the negative impact of postoperative complications on survival in older patients undergoing intensive chemotherapy [50], there is a critical need for prehabilitation during NAT to benefit older patients with advanced esophageal cancer. Further clinical studies are needed to examine the impact of age on the effect of prehabilitation during NAT.

### Conclusion

This review demonstrates that prehabilitation during NAT may safely maintain physical fitness, improve tolerance to NAT, and reduce postoperative complications in patients with esophageal or gastroesophageal junction cancer. However, although the potential usefulness of prehabilitation was demonstrated, only two RCTs were eligible for inclusion, which suggested that the high-quality evidence was currently lacking. In the future, the benefit of prehabilitation during NAT and the mechanisms through which prehabilitation improves clinical outcomes should be examined in RCTs with sufficient sample sizes.

### Supplementary Information

Below is the link to the electronic supplementary material.

Supplementary file1 (DOCX 539 KB)
