# Supplementary material for: Effectiveness of prehabilitation during neoadjuvant therapy for patients with esophageal or gastroesophageal junction cancer: a systematic review
Source: Esophagus. 2024 Feb 27;21(3):283–97. doi: 10.1007/s10388-024-01049-9 (PMC11199248; doi:10.1007/s10388-024-01049-9)
Supplement: Supplementary file 1 — Supplementary file1 (DOCX 539 KB) [file 10388_2024_1049_MOESM1_ESM.docx]

**Online Resource**

**Online Resource 1: search strategy**

Appendix 1: CENTRAL search strategy

Participant Keywords: ([mh "Esophageal Neoplasms"] OR esophageal:ti,ab OR oesophageal:ti,ab OR esophagus:ti,ab OR oesophagus:ti,ab OR gastro-esophageal:ti,ab OR gastro-oesophageal:ti,ab OR gastroesophageal:ti,ab OR esophagogastric:ti,ab) AND ([mh Chemoradiotherapy] OR chemoradiotherapy:ti,ab OR chemo-radiotherapy:ti,ab OR chemoradiotherapies:ti,ab OR chemo-radiotherapies:ti,ab OR chemoradiation:ti,ab OR chemotherapy:ti,ab OR chemotherapies:ti,ab OR radiochemotherapy:ti,ab OR radio-chemotherapy:ti,ab OR radiochemotherapies:ti,ab OR radio-chemotherapies:ti,ab OR adjuvant:ti,ab OR neoadjuvant:ti,ab OR neo‐adjuvant:ti,ab OR [mh "Neoadjuvant Therapy"] OR "multimodal therapy":ti,ab)

Intervention Keywords: ("Physical therapy":ti,ab OR physiotherapy:ti,ab OR "exercise therapy":ti,ab OR prehabilitation:ti,ab OR "preoperative training":ti,ab OR "preoperative exercise training":ti,ab OR "inspiratory muscle training":ti,ab OR "inspiratory training":ti,ab OR [mh "Preoperative Care"] OR [mh "Preoperative Exercise"] OR preconditioning:ti,ab OR [mh exercise] OR Aerobic*:ti,ab OR ("physical" NEXT activit*):ti,ab OR "physical training":ti,ab OR ergomet*:ti,ab OR bicycle:ti,ab OR treadmill:ti,ab OR walk*:ti,ab OR swim*:ti,ab OR cycl*:ti,ab OR run*:ti,ab OR yoga:ti,ab OR "tai chi":ti,ab OR pilates:ti,ab OR IMT:ti,ab OR "preoperative care":ti,ab OR "respiratory training":ti,ab OR "resistance training":ti,ab OR ("muscle" NEXT strength*):ti,ab)

Study design Keywords: NOT ([mh animals] NOT [mh humans])

Appendix 2: PubMed search strategy

Participant Keywords:

(Esophageal Neoplasms[mh] OR esophageal[tiab] OR oesophageal[tiab] OR esophagus[tiab] OR oesophagus[tiab] OR gastro-esophageal[tiab] OR gastro-oesophageal[tiab] OR gastroesophageal[tiab] OR esophagogastric[tiab]

AND

(Chemoradiotherapy[mh] OR chemoradiotherapy[tiab] OR chemo-radiotherapy[tiab] OR chemoradiotherapies[tiab] OR chemo-radiotherapies[tiab] OR chemoradiation[tiab] OR chemotherapy[tiab] OR chemotherapies[tiab] OR radiochemotherapy[tiab] OR radio-chemotherapy[tiab] OR radiochemotherapies[tiab] OR radio-chemotherapies[tiab] OR adjuvant[tiab] OR neoadjuvant[tiab] OR neo‐adjuvant[tiab] OR Neoadjuvant Therapy[mh] OR multimodal therapy[tiab])

Intervention Keywords:

Physical therapy[tiab] OR physiotherapy[tiab] OR exercise therapy[tiab] OR prehabilitation[tiab] OR preoperative training[tiab] OR preoperative exercise training[tiab] OR inspiratory muscle training[tiab] OR inspiratory training[tiab] OR Preoperative Care[mh] OR Preoperative Exercise[mh] OR preconditioning[tiab] OR exercise[mh] OR Aerobic*[tiab] OR physical activit*[tiab] OR physical training[tiab] OR ergomet*[tiab] OR bicycle[tiab] OR treadmill[tiab] OR walk*[tiab] OR swim*[tiab] OR cycl*[tiab] OR run*[tiab] OR yoga[tiab] OR tai chi[tiab] OR pilates[tiab] OR IMT[tiab] OR preoperative care[tiab] OR respiratory training[tiab] OR resistance training[tiab] OR muscle strength*[tiab]

Study design Keywords:

NOT (animals[mh] NOT humans[mh])

Appendix 3: CINAHL search strategy

Participant Keywords: ((MH "Esophageal Neoplasms+") OR (TI esophageal OR AB esophageal) OR (TI oesophageal OR AB oesophageal) OR (TI esophagus OR AB esophagus) OR (TI oesophagus OR AB oesophagus) OR (TI gastro-esophageal OR AB gastro-esophageal) OR (TI gastro-oesophageal OR AB gastro-oesophageal) OR (TI gastroesophageal OR AB gastroesophageal) OR (TI esophagogastric OR AB esophagogastric)) AND ((MH Chemoradiotherapy+) OR (TI chemoradiotherapy OR AB chemoradiotherapy) OR (TI chemo-radiotherapy OR AB chemo-radiotherapy) OR (TI chemoradiotherapies OR AB chemoradiotherapies) OR (TI chemo-radiotherapies OR AB chemo-radiotherapies) OR (TI chemoradiation OR AB chemoradiation) OR (TI chemotherapy OR AB chemotherapy) OR (TI chemotherapies OR AB chemotherapies) OR (TI radiochemotherapy OR AB radiochemotherapy) OR (TI radio-chemotherapy OR AB radio-chemotherapy) OR (TI radiochemotherapies OR AB radiochemotherapies) OR (TI radio-chemotherapies OR AB radio-chemotherapies) OR (TI adjuvant OR AB adjuvant) OR (TI neoadjuvant OR AB neoadjuvant) OR (TI neo‐adjuvant OR AB neo‐adjuvant) OR (MH "Neoadjuvant Therapy+") OR (TI "multimodal therapy" OR AB "multimodal therapy"))

Intervention Keywords: ((TI "Physical therapy" OR AB "Physical therapy") OR (TI physiotherapy OR AB physiotherapy) OR (TI "exercise therapy" OR AB "exercise therapy") OR (TI prehabilitation OR AB prehabilitation) OR (TI "preoperative training" OR AB "preoperative training") OR (TI "preoperative exercise training" OR AB "preoperative exercise training") OR (TI "inspiratory muscle training" OR AB "inspiratory muscle training") OR (TI "inspiratory training" OR AB "inspiratory training") OR (MH "Preoperative Care+") OR (MH "Preoperative Exercise+") OR (TI preconditioning OR AB preconditioning) OR (MH exercise+) OR (TI Aerobic* OR AB Aerobic*) OR (TI "physical activit*" OR AB "physical activit*") OR (TI "physical training" OR AB "physical training") OR (TI ergomet* OR AB ergomet*) OR (TI bicycle OR AB bicycle) OR (TI treadmill OR AB treadmill) OR (TI walk* OR AB walk*) OR (TI swim* OR AB swim*) OR (TI cycl* OR AB cycl*) OR (TI run* OR AB run*) OR (TI yoga OR AB yoga) OR (TI "tai chi" OR AB "tai chi") OR (TI pilates OR AB pilates) OR (TI IMT OR AB IMT) OR (TI "preoperative care" OR AB "preoperative care") OR (TI "respiratory training" OR AB "respiratory training") OR (TI "resistance training" OR AB "resistance training") OR (TI "muscle strength*" OR AB "muscle strength*"))

Study design Keywords: NOT ((MH animals+) NOT (MH humans+))

Appendix 4: Web of Science search strategy

Participant Keywords: (ALL="Esophageal Neoplasms" OR (TI=esophageal OR AB=esophageal) OR (TI=oesophageal OR AB=oesophageal) OR (TI=esophagus OR AB=esophagus) OR (TI=oesophagus OR AB=oesophagus) OR (TI=gastro-esophageal OR AB=gastro-esophageal) OR (TI=gastro-oesophageal OR AB=gastro-oesophageal) OR (TI=gastroesophageal OR AB=gastroesophageal) OR (TI=esophagogastric OR AB=esophagogastric)) AND (ALL=Chemoradiotherapy OR (TI=chemoradiotherapy OR AB=chemoradiotherapy) OR (TI=chemo-radiotherapy OR AB=chemo-radiotherapy) OR (TI=chemoradiotherapies OR AB=chemoradiotherapies) OR (TI=chemo-radiotherapies OR AB=chemo-radiotherapies) OR (TI=chemoradiation OR AB=chemoradiation) OR (TI=chemotherapy OR AB=chemotherapy) OR (TI=chemotherapies OR AB=chemotherapies) OR (TI=radiochemotherapy OR AB=radiochemotherapy) OR (TI=radio-chemotherapy OR AB=radio-chemotherapy) OR (TI=radiochemotherapies OR AB=radiochemotherapies) OR (TI=radio-chemotherapies OR AB=radio-chemotherapies) OR (TI=adjuvant OR AB=adjuvant) OR (TI=neoadjuvant OR AB=neoadjuvant) OR (TI=neo‐adjuvant OR AB=neo‐adjuvant) OR ALL="Neoadjuvant Therapy" OR (TI="multimodal therapy" OR AB="multimodal therapy"))

Intervention Keywords: ((TI="Physical therapy" OR AB="Physical therapy") OR (TI=physiotherapy OR AB=physiotherapy) OR (TI="exercise therapy" OR AB="exercise therapy") OR (TI=prehabilitation OR AB=prehabilitation) OR (TI="preoperative training" OR AB="preoperative training") OR (TI="preoperative exercise training" OR AB="preoperative exercise training") OR (TI="inspiratory muscle training" OR AB="inspiratory muscle training") OR (TI="inspiratory training" OR AB="inspiratory training") OR ALL="Preoperative Care" OR ALL="Preoperative Exercise" OR (TI=preconditioning OR AB=preconditioning) OR ALL=exercise OR (TI=Aerobic* OR AB=Aerobic*) OR (TI="physical activit*" OR AB="physical activit*") OR (TI="physical training" OR AB="physical training") OR (TI=ergomet* OR AB=ergomet*) OR (TI=bicycle OR AB=bicycle) OR (TI=treadmill OR AB=treadmill) OR (TI=walk* OR AB=walk*) OR (TI=swim* OR AB=swim*) OR (TI=cycl* OR AB=cycl*) OR (TI=run* OR AB=run*) OR (TI=yoga OR AB=yoga) OR (TI="tai chi" OR AB="tai chi") OR (TI=pilates OR AB=pilates) OR (TI=IMT OR AB=IMT) OR (TI="preoperative care" OR AB="preoperative care") OR (TI="respiratory training" OR AB="respiratory training") OR (TI="resistance training" OR AB="resistance training") OR (TI="muscle strength*" OR AB="muscle strength*"))

Study design Keywords: NOT (ALL=animals NOT ALL=humans)

Appendix 5: Scopus search strategy

Participant Keywords: TITLE-ABS (“Esophageal Neoplasms” OR esophageal OR oesophageal OR esophagus OR oesophagus OR gastro-esophageal OR gastro-oesophageal OR gastroesophageal OR esophagogastric) AND TITLE-ABS (Chemoradiotherapy OR chemoradiotherapy OR chemo-radiotherapy OR chemoradiotherapies OR chemo-radiotherapies OR chemoradiation OR chemotherapy OR chemotherapies OR radiochemotherapy OR radio-chemotherapy OR radiochemotherapies OR radio-chemotherapies OR adjuvant OR neoadjuvant OR neo‐adjuvant OR “Neoadjuvant Therapy” OR “multimodal therapy”)

Intervention Keywords: TITLE-ABS(“Physical therapy” OR physiotherapy OR “exercise therapy” OR prehabilitation OR “preoperative training” OR “preoperative exercise training” OR “inspiratory muscle training” OR “inspiratory training” OR “Preoperative Care” OR “Preoperative Exercise” OR preconditioning OR exercise OR Aerobic* OR “physical activit*” OR “physical training” OR ergomet* OR bicycle OR treadmill OR walk* OR swim* OR cycl* OR run* OR yoga OR “tai chi” OR pilates OR IMT OR “preoperative care” OR “respiratory training” OR “resistance training” OR “muscle strength*“)

Study design Keywords: NOT (animals AND NOT humans)

Appendix 6: PEDro search strategy

Abstract & Title: neoadjuvant

Appendix 7: ClinicalTrials.gov search strategy

Targeted Search

Intervention/Treatment: prehabilitation

**Online Resource 2: PRISMA 2020 checklist**

| **Section and Topic** | **Item #** | **Checklist item** | **Location where item is reported** |
| --- | --- | --- | --- |
| **TITLE** | | |  |
| Title | 1 | Identify the report as a systematic review. | P1 |
| **ABSTRACT** | | |  |
| Abstract | 2 | See the PRISMA 2020 for Abstracts checklist. | P3 |
| **INTRODUCTION** | | |  |
| Rationale | 3 | Describe the rationale for the review in the context of existing knowledge. | P5, 6 |
| Objectives | 4 | Provide an explicit statement of the objective(s) or question(s) the review addresses. | P6 |
| **METHODS** | | |  |
| Eligibility criteria | 5 | Specify the inclusion and exclusion criteria for the review and how studies were grouped for the syntheses. | P8 |
| Information sources | 6 | Specify all databases, registers, websites, organisations, reference lists and other sources searched or consulted to identify studies. Specify the date when each source was last searched or consulted. | P7 |
| Search strategy | 7 | Present the full search strategies for all databases, registers and websites, including any filters and limits used. | Online Resource 1 |
| Selection process | 8 | Specify the methods used to decide whether a study met the inclusion criteria of the review, including how many reviewers screened each record and each report retrieved, whether they worked independently, and if applicable, details of automation tools used in the process. | P7 |
| Data collection process | 9 | Specify the methods used to collect data from reports, including how many reviewers collected data from each report, whether they worked independently, any processes for obtaining or confirming data from study investigators, and if applicable, details of automation tools used in the process. | P7 |
| Data items | 10a | List and define all outcomes for which data were sought. Specify whether all results that were compatible with each outcome domain in each study were sought (e.g. for all measures, time points, analyses), and if not, the methods used to decide which results to collect. | P8, 9 |
|  | 10b | List and define all other variables for which data were sought (e.g. participant and intervention characteristics, funding sources). Describe any assumptions made about any missing or unclear information. | P8, 9 |
| Study risk of bias assessment | 11 | Specify the methods used to assess risk of bias in the included studies, including details of the tool(s) used, how many reviewers assessed each study and whether they worked independently, and if applicable, details of automation tools used in the process. | P9, 10 |
| Effect measures | 12 | Specify for each outcome the effect measure(s) (e.g. risk ratio, mean difference) used in the synthesis or presentation of results. | P10 |
| Synthesis methods | 13a | Describe the processes used to decide which studies were eligible for each synthesis (e.g. tabulating the study intervention characteristics and comparing against the planned groups for each synthesis (item #5)). | P10 |
|  | 13b | Describe any methods required to prepare the data for presentation or synthesis, such as handling of missing summary statistics, or data conversions. | P10 |
|  | 13c | Describe any methods used to tabulate or visually display results of individual studies and syntheses. | P10 |
|  | 13d | Describe any methods used to synthesize results and provide a rationale for the choice(s). If meta-analysis was performed, describe the model(s), method(s) to identify the presence and extent of statistical heterogeneity, and software package(s) used. | P10 |
|  | 13e | Describe any methods used to explore possible causes of heterogeneity among study results (e.g. subgroup analysis, meta-regression). | P10 |
|  | 13f | Describe any sensitivity analyses conducted to assess robustness of the synthesized results. | P10-11 |
| Reporting bias assessment | 14 | Describe any methods used to assess risk of bias due to missing results in a synthesis (arising from reporting biases). | P11 |
| Certainty assessment | 15 | Describe any methods used to assess certainty (or confidence) in the body of evidence for an outcome. | P9 |
| **RESULTS** | | |  |
| Study selection | 16a | Describe the results of the search and selection process, from the number of records identified in the search to the number of studies included in the review, ideally using a flow diagram. | Fig. 1 P12 |
|  | 16b | Cite studies that might appear to meet the inclusion criteria, but which were excluded, and explain why they were excluded. | Fig. 1 |
| Study characteristics | 17 | Cite each included study and present its characteristics. | Table 2, 3 P12 |
| Risk of bias in studies | 18 | Present assessments of risk of bias for each included study. | Fig. 2 Online Resource 3, 4  P13 |
| Results of individual studies | 19 | For all outcomes, present, for each study: (a) summary statistics for each group (where appropriate) and (b) an effect estimate and its precision (e.g. confidence/credible interval), ideally using structured tables or plots. | Fig. 3 Online Resource 5,6  Table 4 |
| Results of syntheses | 20a | For each synthesis, briefly summarise the characteristics and risk of bias among contributing studies. | P12, 13 |
|  | 20b | Present results of all statistical syntheses conducted. If meta-analysis was done, present for each the summary estimate and its precision (e.g. confidence/credible interval) and measures of statistical heterogeneity. If comparing groups, describe the direction of the effect. | P12, 13 |
|  | 20c | Present results of all investigations of possible causes of heterogeneity among study results. | Fig. 3 |
|  | 20d | Present results of all sensitivity analyses conducted to assess the robustness of the synthesized results. | P14 |
| Reporting biases | 21 | Present assessments of risk of bias due to missing results (arising from reporting biases) for each synthesis assessed. | Online Resource 7 |
| Certainty of evidence | 22 | Present assessments of certainty (or confidence) in the body of evidence for each outcome assessed. | Table 1 |
| **DISCUSSION** | | |  |
| Discussion | 23a | Provide a general interpretation of the results in the context of other evidence. | P16-21 |
|  | 23b | Discuss any limitations of the evidence included in the review. | P21, 22 |
|  | 23c | Discuss any limitations of the review processes used. | P21 |
|  | 23d | Discuss implications of the results for practice, policy, and future research. | P16, 17 |
| **OTHER INFORMATION** | | |  |
| Registration and protocol | 24a | Provide registration information for the review, including register name and registration number, or state that the review was not registered. | P7 |
|  | 24b | Indicate where the review protocol can be accessed, or state that a protocol was not prepared. | P7 |
|  | 24c | Describe and explain any amendments to information provided at registration or in the protocol. | N/A |
| Support | 25 | Describe sources of financial or non-financial support for the review, and the role of the funders or sponsors in the review. | P22 |
| Competing interests | 26 | Declare any competing interests of review authors. | P22 |
| Availability of data, code and other materials | 27 | Report which of the following are publicly available and where they can be found: template data collection forms; data extracted from included studies; data used for all analyses; analytic code; any other materials used in the review. | Online Resource 1 |

**Online Resource 3: Quality assessment of included studies according to the Newcastle-Ottawa Scale for non-RCT studies**

| **Study** | **Selection** |  |  |  |  | **Comparability** |  | **Outcome** |  |  |  | **Total score** |
| --- | --- | --- | --- | --- | --- | --- | --- | --- | --- | --- | --- | --- |
|  | **(1)** | **(2)** | **(3)** | **(4)** |  | **(5)** |  | **(6)** | **(7)** | **(8)** |  |  |
| Zylstra et al. (2022) | ★ | ★ | ☆ | ★ |  | ★★ |  | ☆ | ★ | ★ |  | 7/9 |
| Christensen et al. (2018) | ★ | ★ | ★ | ★ |  | ☆ |  | ☆ | ★ | ★ |  | 6/9 |
| Halliday et al. (2023) | ★ | ★ | ★ | ★ |  | ★★ |  | ★ | ★ | ☆ |  | 8/9 |
| **(1):** Representativeness of the exposed cohort; (**2):** Selection of the non-exposed cohort; **(3):** Ascertainment of exposure; (**4):** Demonstration that outcome of interest was not present at start of study; (**5):** Comparability of cohorts on the basis of the design or analysis; **(6):** Assessment of outcome; (**7):** Was follow-up long enough for outcomes to occur; (**8):** Adequacy of follow up of cohort | | | | | | | | | | | | |

**Online Resource** **4: Methodological quality of before-after studies using the ‘Quality Assessment Tool for Before After (Pre-Post) Studies with No Control Group'**

| **Study** | **(1)** | **(2)** | **(3)** | **(4)** | **(5)** | **(6)** | **(7)** | **(8)** | **(9)** | **(10)** | **(11)** | **(12)** |  | **Total score** |
| --- | --- | --- | --- | --- | --- | --- | --- | --- | --- | --- | --- | --- | --- | --- |
| Ikeda et al.  (2022) | Y | Y | Y | Y | CD | Y | Y | Y | Y | Y | Y | NA |  | Good |
| Halliday et al. (2021) | Y | Y | Y | Y | CD | CD | Y | NR | Y | Y | Y | NA |  | Fair |
| Chmelo et al. (2022) | Y | Y | Y | Y | Y | CD | Y | NR | Y | N | Y | NA |  | Fair |
| Yang et al.  (2021) | Y | Y | Y | Y | NR | CD | Y | NR | Y | Y | Y | NA |  | Fair |
| **(1):** Objective clearly stated; **(2):** eligibility criteria described; **(3):** representative patient population; **(4):** all eligible participants enrolledin study; **(5):** sufficient sample size; **(6):** intervention described; **(7):** outcome measures specified; **(8):** outcome assessors blinded; **(9):** loss to follow-up; **(10):** statistical analysis for pre-to-post changes; **(11):** interruptedtime-series design; **(12):** individual data used for group-level effects.  Abbreviations: **Y:** yes; **N:** no; **NA:** not applicable; **NR:** not reported; **CD:** cannot determine. **Overall quality rating:** good, fair, or poor. | | | | | | | | | | | | | | |

**Online Resource 5. Sensitivity analysis of the effect of prehabilitation on exercise capacity**


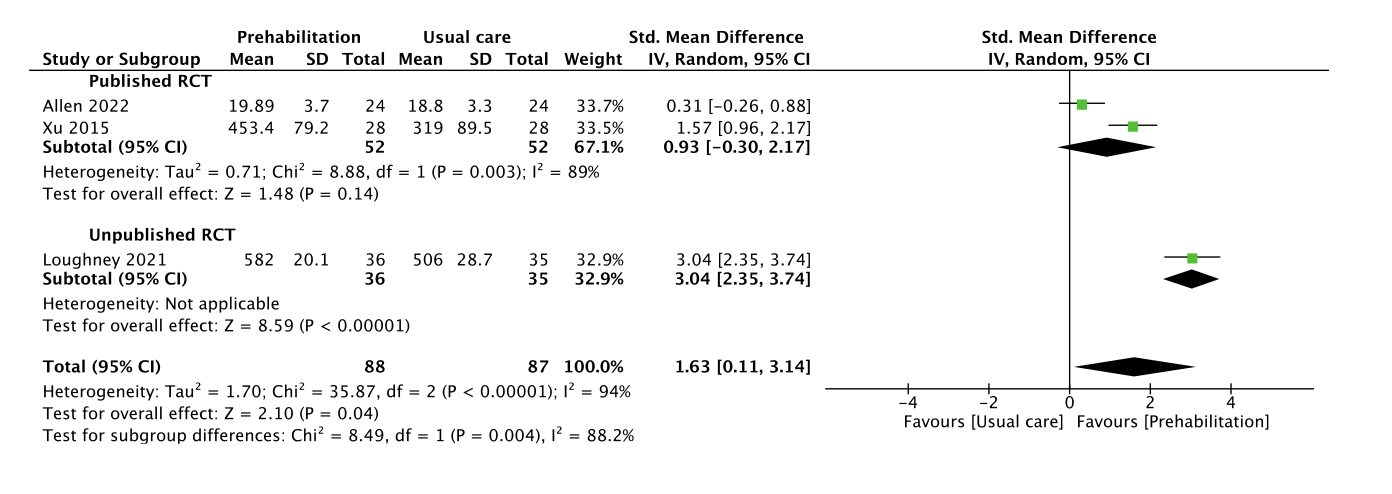


Forest plot using published randomized controlled trial (RCT) and unpublished literature

Std., standard; 95% CI, 95 % confidence interval

Cases with I^2^ > 50% are considered substantially heterogeneous

**
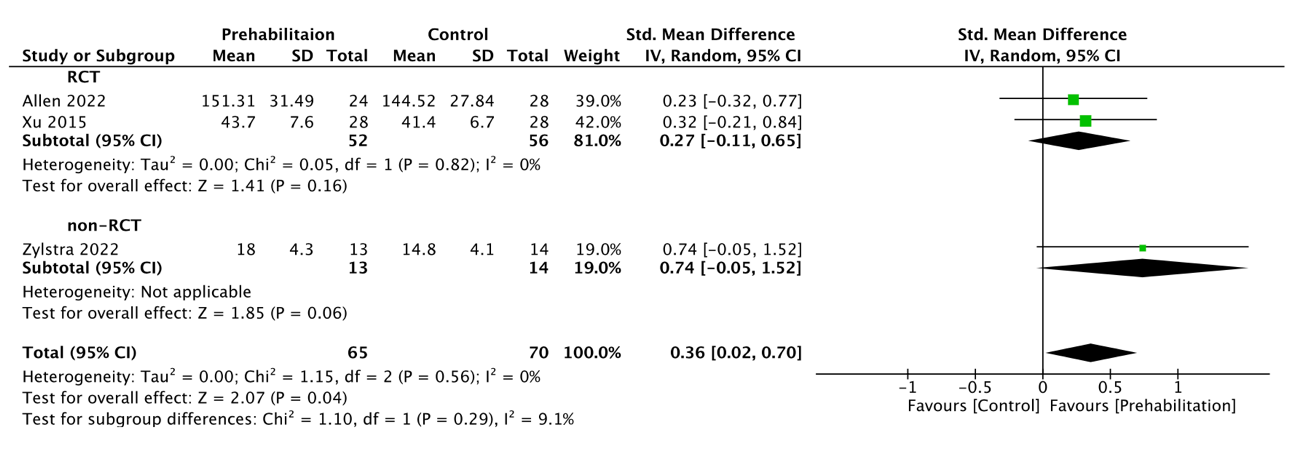
Online Resource 6. Sensitivity analysis of the effect of prehabilitation on skeletal muscle mass**

Forest plot using randomized controlled trials (RCTs) and non-RCTs

Std., standard; 95% CI, 95 % confidence interval

Cases with I^2^ > 50% are considered substantially heterogeneous

**Online Resource 7. Funnel plot of studies evaluating the publication bias**


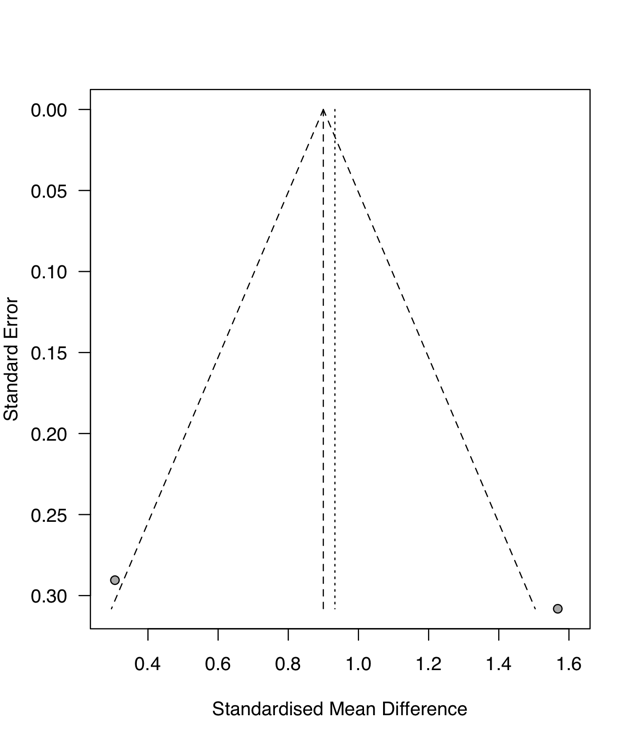

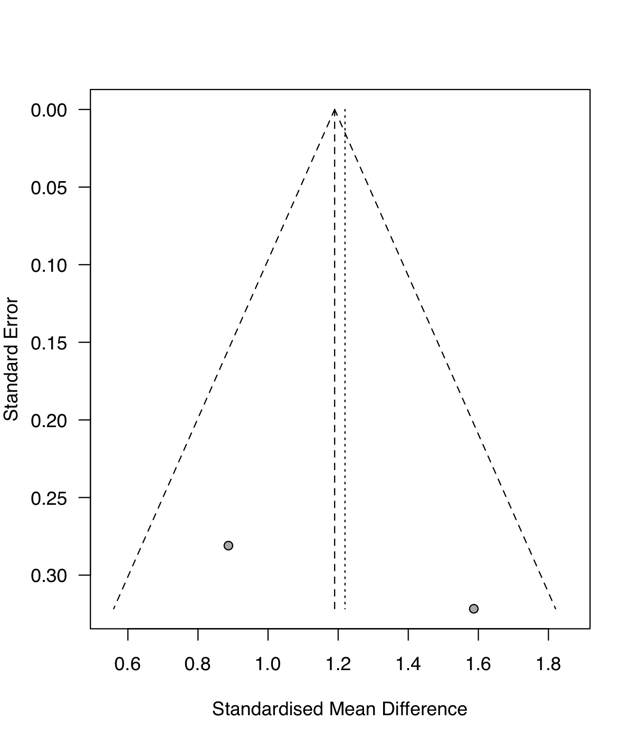


**b**

**a**


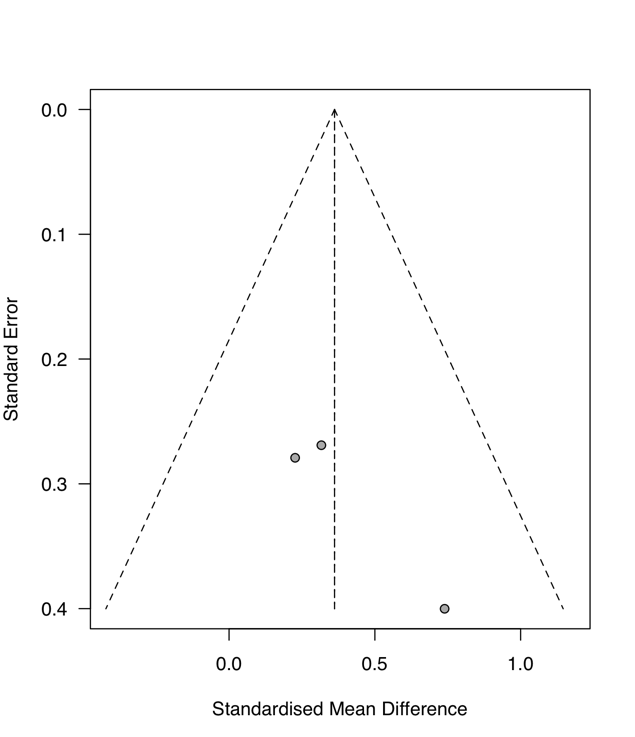

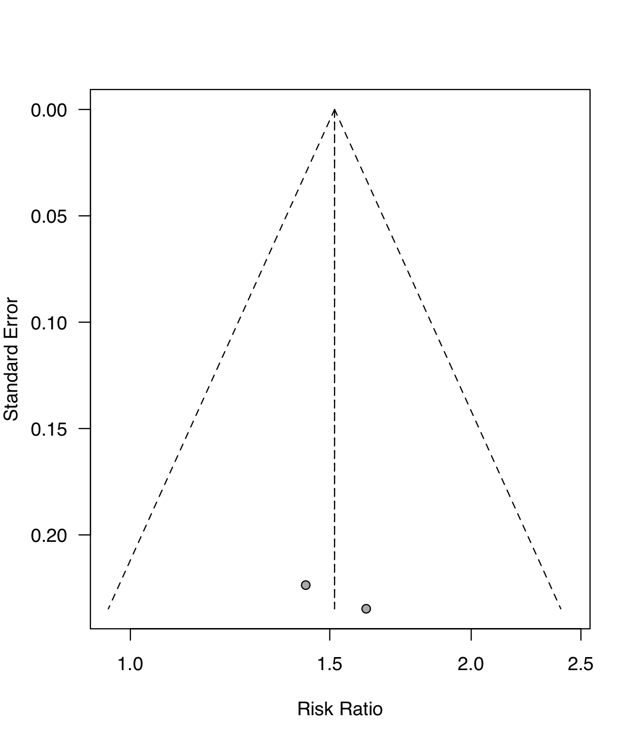


**d**

**c**


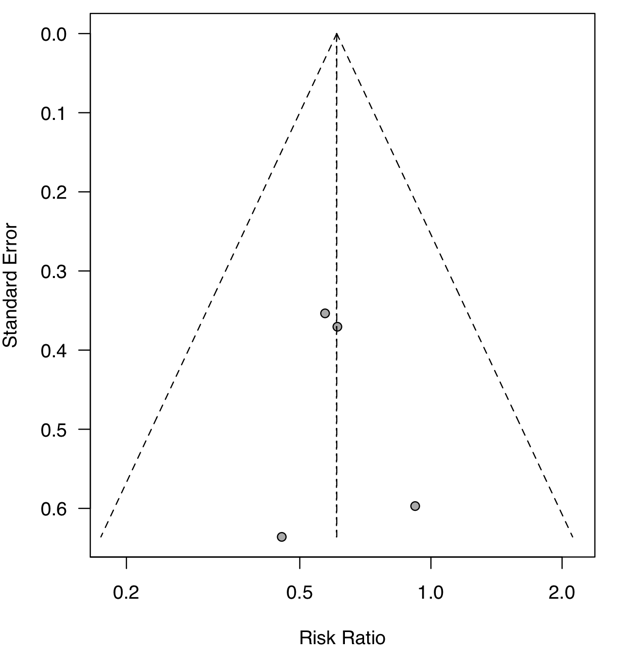


**e**

Funnel plots to assess publication bias in studies examining the effect of prehabilitation during NAC on exercise capacity (a), grip strength (b), skeletal muscle mass (c), tolerance to NAT (d) and postoperative complications (e).

Funnel plot figures in (a), (b), and (d) include only RCTs.

Funnel plot figures in (c) and (e) include RCTs and non-RCTs.

Journal: Esophagus

Title: Effectiveness of prehabilitation during neoadjuvant therapy for patients with esophageal or gastroesophageal junction cancer: a systematic review and meta-analysis

Tomohiro Ikeda, MSc^1^, Shusuke Toyama, MSc^2^, Tsuyoshi Harada, MSc^3,4^, Kazuhiro Noma, PhD^5^, Masanori Hamada, PhD^1^, Takashi Kitagawa, PhD^6^
^1^Department of Rehabilitation Medicine, Okayama University Hospital, 2-5-1 Shikatacho, Kita-ku, Okayama 700-8558, Japan
^2^Department of Rehabilitation, Tagami Hospital, 2-14-15 tagami, Nagasaki, Japan

^3^Department of Rehabilitation Medicine, National Cancer Center Hospital East, 6-5-1 Kashiwa, Chiba, Japan

^4^Department of Rehabilitation Medicine, Keio University Graduate School, 35 Shinanomachi, Shinjuku-ku, Tokyo Japan

^5^Department of Gastroenterological Surgery, Graduate School of Medicine, Dentistry and Pharmaceutical Sciences, Okayama University, 2-5-1 Shikatacho, Kita-ku, Okayama, Japan

^6^Department of Physical Therapy, School of Health Sciences, Shinshu University, 3‑1‑1 Asahi, Matsumoto, Nagano, Japan

**Correspondence:** Tomohiro Ikeda

E-mail address: pn9375yc@s.okayama-u.ac.jp

Telephone number: +81-86-235-7752

Fax number: +81-86-235-7751
